# Exfoliation of MoS_2_ Quantum Dots: Recent Progress and Challenges

**DOI:** 10.3390/nano12193465

**Published:** 2022-10-04

**Authors:** Luqman Ali, Fazle Subhan, Muhammad Ayaz, Syed Shams ul Hassan, Clare Chisu Byeon, Jong Su Kim, Simona Bungau

**Affiliations:** 1 Department of Physics, Yeungnam University, Gyeongsan 38541, Korea; 2 School of Mechanical Engineering, Kyungpook National University, Daegu 41566, Korea; 3 Department of Physics, University of Lakki Marwat, Lakki Marwat 28420, Pakistan; 4 Department of Pharmacy, Faculty of Biological Sciences, University of Malakand, Chakdara 18000, Pakistan; 5 Shanghai Key Laboratory for Molecular Engineering of Chiral Drugs, School of Pharmacy, Shanghai Jiao Tong University, Shanghai 200240, China; 6 Department of Natural Product Chemistry, School of Pharmacy, Shanghai Jiao Tong University, Shanghai 200240, China; 7 Department of Pharmacy, Faculty of Medicine and Pharmacy, University of Oradea, 410028 Oradea, Romania

**Keywords:** 2D materials, graphene, MoS_2_, quantum dots, exfoliation

## Abstract

Although, quantum dots (QDs) of two-dimensional (2D) molybdenum disulfide (MoS_2_) have shown great potential for various applications, such as sensing, catalysis, energy storage, and electronics. However, the lack of a simple, scalable, and inexpensive fabrication method for QDs is still a challenge. To overcome this challenge, a lot of attention has been given to the fabrication of QDs, and several fabrication strategies have been established. These exfoliation processes are mainly divided into two categories, the ‘top-down’ and ‘bottom-up’ methods. In this review, we have discussed different top-down exfoliation methods used for the fabrication of MoS_2_ QDs and the advantages and limitations of these methods. A detailed description of the various properties of QDs is also presented.

## 1. Introduction

Although 2D materials have been theorized since the 1940s [1] and are the most expansively studied and broadly considered materials because of the exotic physical phenomena of heat and charge transfer in a plane [2]. The discovery of graphene [3] has re-raised a significant attraction in the scientific community. This driven interest of the research community unveiled numerous 2D layered materials like hexagonal Boron Nitride (hBN), borophene (2D boron), layered transition metal chalcogenides (LTMDs), perovskites, and several other materials listed in Table 1. 2D materials e.g., graphene, hBN, LTMDs, perovskites, and so on, are widely used in many fields of applications. These applications include stretchable electronic wearables, flexible displays, fast and light chips for 5G communications, space and military equipment, environment, food and health monitoring, photovoltaics, energy storage devices, and so on.

**Figure 1:**
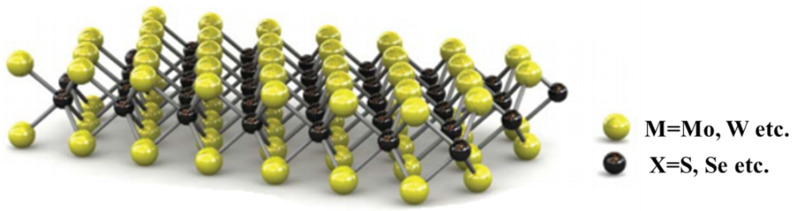
M and X-atoms arranged in a monolayer of LTMDs. Reproduced with permission from [4]. Springer International Publishing Switzerland, 2011.

Among the 2D materials, LTMDs have shown great potential for several applications. 2D LTMDs are layered materials of the stoichiometry MX2, where the metal atoms (M = Mo, W, etc.) are packed in between the chalcogen’s atoms (X = S, Se, Te, etc.) making the single layer with the form X–M–X, as pictured in Figure 1. These single layers then stack around by weak van der Waal’s forces to make three-dimensional (3D) bulk structures. 2D LTMDs intrinsically exhibit exceptional physiochemical properties.

Depending on the composition of chalcogens and transition metals, more than 40 different LTMDs can occur. Because of the oxidation state and coordination of the atoms, they can be of metallic, semi-metallic, or semiconducting nature [5,6]. Molybdenum disulfide (MoS_2_) is one of the most important and studied materials of the 2D LTMDs family. Unlike metallic graphene, with extraordinarily high carrier mobilities of about ~15,000 cm^2^/Vs (at room temperature) and zero band gap, MoS_2_ has the advantage of the direct bandgap and semiconducting properties with good mobilities in specific conditions. For instance, at room temperature, the carrier’s mobility of MoS_2_ on a Si/SiO_2_ substrate (with Sc electrical contacts) reaches 700 cm^2^V^−1^s^−1^ on SiO_2_/Si substrate, and on Si/BN substrate it is 33–151 cm^2^V^−1^s^−1^ [7,8]. In addition to outstanding electrical mobility like graphene, MoS_2_ also possesses good mechanical flexibility and strength. For example, exceptionally large values of Young’s modulus (i.e., E ~0.33 ± 0.07 TPa) [9] for suspended few-layered MoS_2_ flakes and high in-plane stiffness of about ~180 ± 60 Nm^−1^ with E ~270 ± 100 GPa for the MoS_2_ monolayer [10] have been reported. These reports depict that a single layer of MoS_2_ is mechanically more strong than stainless steel (E = 204 GPa) and graphene-oxide (E = 207 GPa) [11], which is due to defect-free structure, highly crystalline nature, and the absence of stacking faults in the one-atom-thick LTMDs. Monolayered MoS_2_ presents drastically diverse properties than its bulk counterpart. As single-layer MoS_2_ absorbs only about 10% of the light incident with energy higher than the bandgap [12]. The photoluminescence (PL) intensity of the single layer MoS_2_, increases 1000-times with a relatively low photoluminescence quantum yield (PLQY) (~0.4%) as compared to the bulk [13]. The PLQY of the single MoS_2_ can be significantly increased (~< 95%), by eradicating the energy traps which cause the non-radiative recombination [14]. Single-layer MoS_2_ shows almost 100 times lower thermal conductivity (of about ~35 Wm^−1^K^−1^) in comparison to graphene [15].

For potential exploitation of these mesmerizing characteristics of MoS_2_, it is needed to be exfoliated into monolayer nanosheets. Owing to the weak interlayer van der Waal’s interactions, bulk MoS_2_ can be easily exfoliated monolayer nanosheets which are further broken into ultra-small QDs. Being an intrinsic LTMD material, MoS_2_ QDs possess both the characteristics of an LTMD and QDs. Several exfoliation techniques like mechanical exfoliation, lithium ion-intercalation, electrochemical exfoliation, electro-Fenton exfoliation, laser ablation, and cryo-mediated exfoliation [16,17,18,19,20,21,22,23,24,25,26] are commonly used. Almost all of these exfoliation processes are multi-step procedures where bulk MoS_2_ is exfoliated into monolayer/few-layer sheets and the sheets are broken into small QDs. The exfoliation strategies are discussed in detail in Section 3.4.

Although a number of useful reviews are present on the exfoliation of MoS_2_ QDs, they only focused on the reported exfoliation strategies in the literature and do not discuss QDs and their unique properties. Also, there is no discussion on some of the very recently developed approaches. In this review, we have discussed what QDs are and why are they so special. Along with this detailed discussion on QDs, we also have included recent approaches, such as laser ablation and cryo-mediated exfoliation methods, which are not discussed earlier. A comparison of the advantages and limitations of the commonly used MoS_2_ exfoliation techniques is also presented.

## 2. MoS_2_

**Figure 2:**
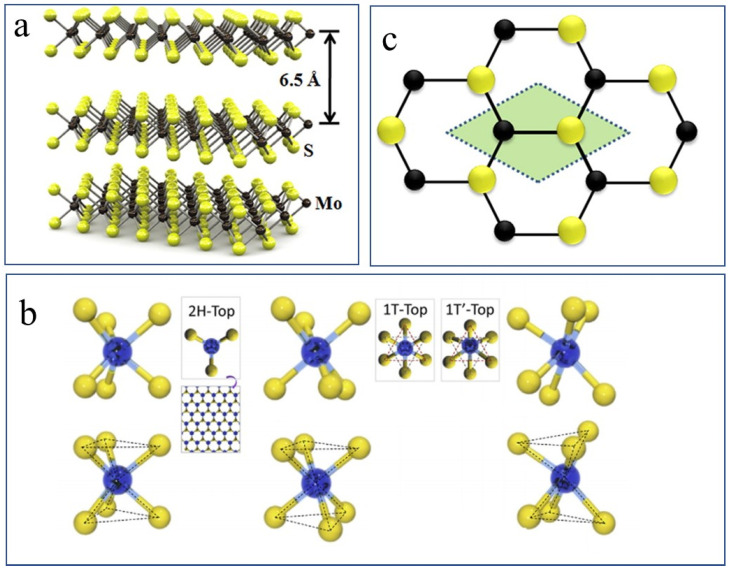
(**a**) Arrangement of S-Mo-S layers in 3D MoS_2_. Where (**b**) and (**c**); show the top view of the MoS_2_ unite cell and the honeycomb lattice of 2H-MoS_2_ crystal, respectively. Reproduced with permissions from [34]. American Chemical Society, 2013.

MoS_2_ is a representative and the most studied material of the LTMDs family. The monolayer structure of MoS_2_ has Mo atoms at M-location sandwiched between the S atoms at the X-location, to form a single layer of MoS_2_. The single layers are vertically stacked and held together through weakly interacting van der Waal’s forces, forming 3D bulk MoS_2_, as shown in Figure 2a.

### 2.1. Crystal Structure

Owing to the atoms’ arrangements in 3D bulk MoS_2_, it possesses three different crystal structures named trigonal-prismatic (hexagonal, 2H), octahedral (tetragonal, 1T), and the distorted-phase (1T’) described in Figure 2b. As evident from the hexagonal symmetry (top-view in Figure 2b of a single MoS_2_ molecule, in the 2H-phase, every Mo-atom branches out to six S-atoms making a tetrahedron (dark-dashed lines) each in the +z and −z dimensions. Consequently, the S–Mo–S arrangement along the vertical or z-direction is termed a monolayer. The weak van der Waal’s (vdW) forces, between the S–S atoms of two layers, make it possible to exfoliate the bulk MoS_2_ into monolayers by applying external mechanical forces.

In the 1T-phase, one of the two trigonal S-layers (top and bottom) in the same S–Mo–S layer has rotated through 180° (forming the so-called trigonal antiprism structure), thus resulting in a hexagonal arrangement of the S-atoms with Mo at the center of the hexagon. The top view of the 1T-phase is also pictured in Figure 2b. Further distortion of the Mo-atoms results in the formation of the distorted 1T’-phase [27,28], causing the re-location of the S-atoms with amended distances of the S-S atoms in the vertical z-direction. The lattice unit cell of the 2H-MoS_2_ crystal, the shaded area in Figure 2c, contains 3 atoms (one Mo and two S atoms) where due to the tri-layered structure the other S-atom cannot be seen here. There is a separation of 3.09 Å between the in-plane S-atoms (S–S bond length or the in-plane lattice constant) and the Mo–S bond length is 2.39 Å [29]. The vertical distance between two S-atoms (of the same layer) is 3.11 Å [30], whereas the interlayer spacing (the vertical distance between the S atoms of two consecutive layers) is 6.5 Å [31], shown in Figure 2a. Naturally, MoS_2_ can be found as a combination of two stable polytypes, i.e., the more stable and more abundant 2H-MoS_2_ (point symmetry group: D_6h_) and the rhombohedral modification 3R-MoS_2_, which can be transformed into 2H-MoS_2_ by heat treatment [32,33]. Most of the exfoliation-related studies include the thermodynamically more stable and relatively abundant 2H-MoS_2_ as the precursor bulk material.

### 2.2. Properties

The distinctive layered structure of MoS_2_, as discussed in the previous section, lends some exciting properties such as a definite bandgap, semiconducting nature, mechanical strength, and good electrical and thermal mobilities to it. In this section, we will try to give a comprehensive description of these exciting properties and characteristics of MoS_2_.

**Figure 3:**
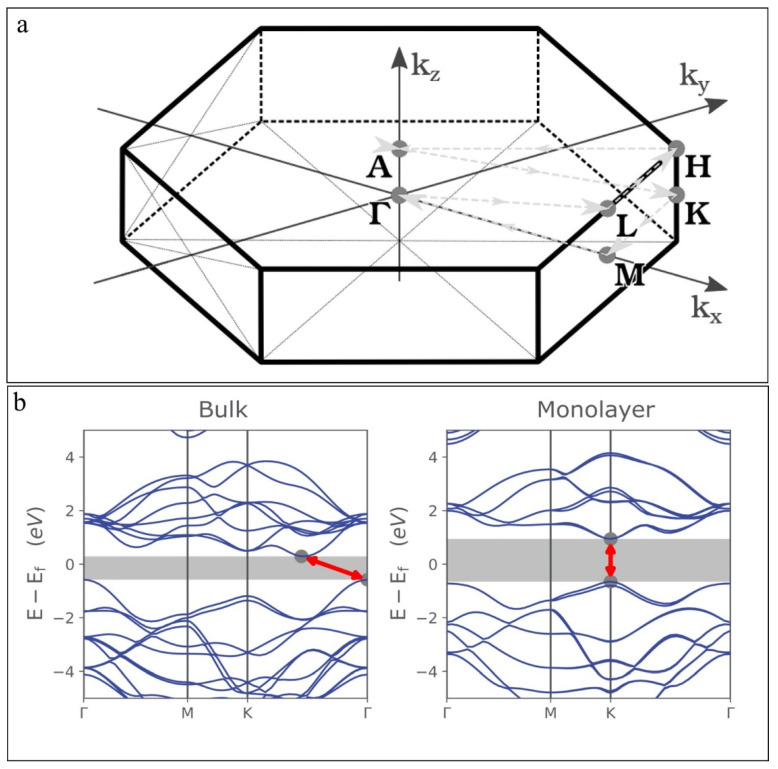
(**a**) First Brillouin zone of 2H-MoS_2_. Reproduced with permissions from [38]. AIP Publishing, 2016. (**b**) Energy band structure diagrams of the bulk and monolayer MoS_2_. Reprinted with permissions from [39]. Ossila, (accessed on 24 September 2022).

The first Brillouin zone (BZ) (shaded grey in Figure 3a) and the corresponding high-symmetry points (i.e., **Γ** point (at k = 0), **M**, **K,** and **H,** etc.) are shown in Figure 3a. In the bulk form, MoS_2_ shows a semiconducting behavior with a significant tunable band-gap (indirect) of ~1.29 eV unlike graphene [13], which is increased to ~1.9 eV (direct) by reducing the thickness of MoS_2_ or transforming it into a single layer. The corresponding band-gap diagrams, of the single-layer and multi-layered bulk MoS_2_ predicted by the first-principles study, are given in Figure 3b. It is clear from Figure 3b, where the effect of layer confinement on the electronic band structure is shown, that the bulk MoS_2_ has a band gap at **Γ**-point in the valance band and midway between the **Γ** and **K** points in the conduction band, which shifts to the **K**-point as the MoS_2_ thickness is reduced to monolayer. The bonding in bulk or single-layer MoS_2_ can explain this bandgap transformation (indirect-direct) with decreasing number of layers or thickness. The density of states (DOS) data shows that MoS_2_ has a filled valance band composed of the d_z_^2^ orbital and a conduction band composed of the degenerate d_x_^2^_−y_^2^ and d_xy_ orbitals [35,36,37]. The valance band overlaps with the p_z_ and the conduction band overlaps with the empty, antibonding p_z_ orbital of the S atoms. The conduction and valance bands of MoS_2_ are primarily composed of the d_x_^2^_−y_^2^ and d_xy_ orbitals of Mo atoms at the K-point, d orbitals of the Mo atoms, and p_z_ orbitals of the S atoms at the **Γ**-point. Because of the van der Waals interactions between the S atoms of two layers, the S atoms experience strong interactions than the Mo atoms. The bonding ascribed to the p_z_ orbitals of the S atoms weakens and consequently, the gap near the **Γ**-point widens on decreasing the thickness of the bulk MoS_2_ to monolayer. On the other hand, the band structure at the K-point is slightly affected because the band structure is primarily due to the in-plane bonding between the Mo atoms. Smaller MoS_2_ QDs have larger bandgaps because the bonding due to the d_x_^2^_−y_^2^ and d_xy_ orbitals of the Mo atoms weakens as the lateral dimensions of the MoS_2_ QDs reduce.

**Figure 4:**
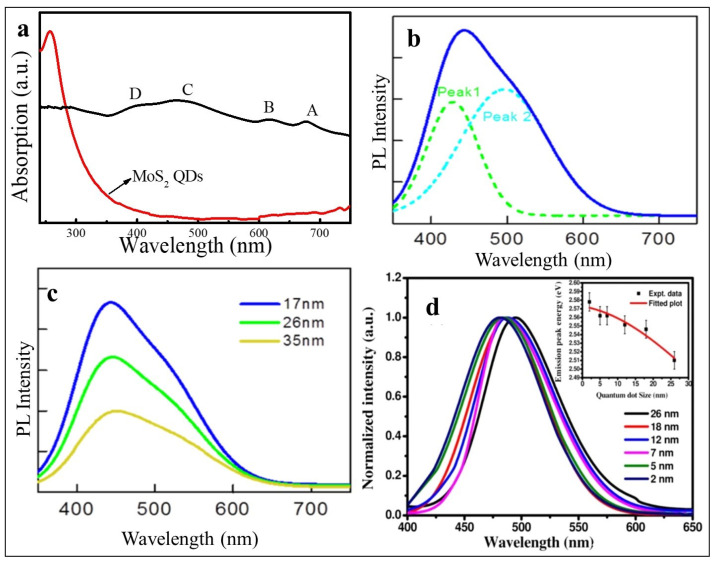
(**a**) UV–Vis absorption of bulk and QDs of MoS_2_. Reproduced with permissions from [25]. Springer Nature, 2019. PL of MoS_2_ QDs; (**b**) size ~17 nm, λ_exc_ = 325 nm; (**c**) sizes of ~17 nm, 26 nm and 35 nm with λ_exc_ = 325 nm. Reproduced with permissions from [42]. IOP Publishing, 2020; and (**d**) PL spectra of different sized MoS_2_ QDs. Reproduced with permissions from [46]. Springer Nature, 2016.

Optical properties such as UV–Vis absorption and PL are widely used to study the quantum confinement effects of the MoS_2_ QDs. The absorption spectrum of the bulk MoS_2_ has four characteristic peaks at 677 nm, 616 nm, 465 nm, and 405 nm labeled A–D, as shown in Figure 4a. The peaks at 616 nm and 677 nm (A and B respectively) are because of the transitions from the split valence band to the conduction band at the **K**- point whereas the C and D peaks at 405 nm and 465 nm can be assigned to the transitions between the split valance and conduction bands at the **M**-point of Brillion zone [40]. The origin of the energy splitting of the peaks is spin-orbit and interlayer coupling, where the inter-layer coupling reduces as the number of layers is reduced [41,42]. The C and D peaks also explain a commonly observed band nesting phenomenon in the LTMDs caused by the Van Hove singularities, which result in the joint density of states and wide band gaps of LTMDs [43]. These four characteristic peaks disappeared in the spectra of the MoS_2_ QDs and only one peak was observed in the UV region (λ ≈ 270 nm) which was attributed to the excitonic features of the QDs [44]. Furthermore, a strong blue shift is observed in the UV–Vis absorption of the prepared QDs compare to bulk MoS_2_ attributed to the quantum confinement effect and edge effect as the lateral sizes of the majority of the prepared QDs are in the 1–5 nm range, and the interlayer coupling is absent [16,25,45].

MoS_2_ QDs results in a broader PL spectrum where the intensities and position of the emission peaks change with change in lateral sizes. Figure 4b shows a typical PL spectrum of MoS_2_ QDs, deconvoluted into two overlapping peaks at ~420 nm and 500 nm [42], where the 420 nm peak is due to the transitions between the quantized energy levels, and the peak at 500 nm is a result of the transitions originated by defects. PL spectra of the MoS_2_ QDs of three different sizes (17 nm, 26 nm, and 35 nm) are shown in Figure 4c, revealing the dependence of the intensities of the emission PL peaks on the size of the QDs. The intensity of the emission peak decreases with an increase in the size of the QDs. Similarly, the PL spectrum in Figure 4d shows that for MoS_2_ QDs with sizes ranging from ~ 2 to 26 nm the emission PL peak shifts from ~480–500 nm because of the quantum confinement effect [46]. Vacuum ultra-violet (VUV) excitation luminescence spectroscopy is another influential tool to investigate the quantum confinement effects of wide band gap materials [47].

## 3. What Are Quantum Dots?

**Figure 5:**
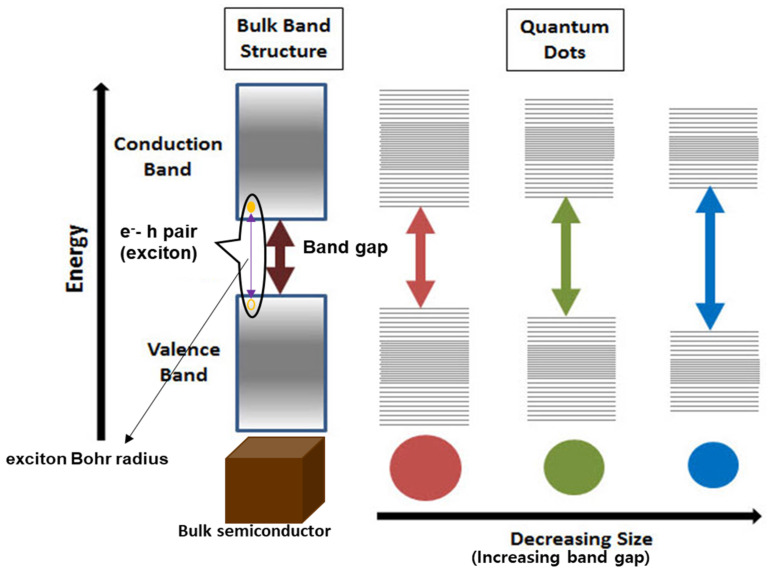
Effect of QDs’ size on their Band gaps.

The widespread and extensive progress in nanotechnology, in the current years, has prompted a series of emerging nanostructures (2D, one-dimensional: 1D, and zero-dimensional: 0D) of 2D materials. These exotic new materials include thin films [27,28], nanorods (NRs) [48,49] and quantum dots (QDs) [16]. Amongst these emerging nanostructures, QDs have shown some matchless and incomparable optical and electronic characteristics making them the most striking and wonderful nanostructures. These amazing characteristics of the QDs are neither offered from the isolated molecules nor bulk solids [50,51,52] and therefore the scientific and engineering communities are equally fascinated by the potential opportunities that arise from the QDs. The recent hype in interest towards LTMDs is all because their tunable edge effects emerged as a result of reducing their planar dimensions below 100 nm [53]. The emergence of new potential properties by size miniaturing has paved the path to the fabrication of QDs. QDs are often defined as the 0D nanocrystals of semiconductors with average radii ranging ~2–10 nm (10–50 atoms) and confined along all three dimensions [54,55]. QDs can also be referred to as semiconductor nanocrystals or particles with physical dimensions comparable to or smaller than the excitonic Bohr radius [56]. An exciton is the bound state of an electron, and a hole is created when a light photon excites the electron from the valance band to the conduction band and thus creates a space (or hole) in the valance band. Electrostatic coulomb force then keeps the electron-hole pair bonded making an exciton. The greatly reduced sizes and spatial confinement along all dimensions of the QDs cause quantum confinement (shown in Figure 5) and edge effects, large surface area-to-volume ratios, and high in-plane transport abilities that greatly enhance their electrocatalytic activity and photoluminescence (PL) quantum yield [57,58] and give them the exotic new catalytic and optoelectronic properties.

### 3.1. Elimination of van der Waal’s Forces

As discussed, bulk layered materials such as graphite and LTMDs consist of planes of covalently and ionic bonded atoms [59] that are stacked to each other by weak van der Waal’s (vdW) forces. These weak interactions are easily dominatable with applying external forces and hence the 3D materials break into monolayer/few-layer graphite and LTMDs. Thus, these weak antistrophic bonding or vdW interactions make the bulk layered materials weak [60]. In comparison, the covalent and ionic bonds holding the atoms together in the single layers are very strong. Since a single layer of atoms only have strong covalent interactions, and thus the removal of these weak links (the vdW interactions) could be a reason that makes the monolayer/few-layer materials very strong as compared to the bulk multilayered materials.

### 3.2. Large Surface Area-to-Volume Ratios

Surface area-to-volume or specific surface area ratios of a material increase as it breaks into smaller pieces and hence QDs take greater surface area-to-volume ratios as compared to their 3D bulk counterparts. It defines how much of the material is exposed to its environment and thus gives the limit of the interaction of a material with its environment. Due to the enlarged surface area-to-volume ratios, the reactivity of the material increases with a reduction in size [42]. Because of the increased reactivity, their catalytic activity also enhances which is beneficial in the applications depending on the catalytic activity of the QDs such as hydrogen evolution reactions (HERs) and colorimetric sensing applications of QDs of 2D materials.

### 3.3. Quantum Confinement Effect

The electronic band structure of a material describes the motion of electrons in the material and thus decides the different characteristics and properties that the material has. The electronic band structure is basically due to the periodic nature of the crystal structures of the materials. When a material breaks into 2D planes or is reduced along one dimension, this periodicity also changes along that dimension and hence changes the band structure. In this modified band structure, the electrons are now restricted to moving in two directions, which is called the quantum confinement effect. This quantum confinement effect then results in the extremely high conductivity of graphene and increased band gap of monolayer MoS_2_ [42,61].

Because of these enhanced and tunable optoelectronic and catalytic properties, QDs are extensively used in light-emitting, photovoltaic and bioimaging, and biosensing devices [62,63,64,65,66]. QDs have shown promising performance in the field of light-emitting diodes “quantum dot light-emitting diodes” with visibly more accurate and outstanding colors and bright emissions in the visible and infrared regions. Optical properties of QDs i.e., absorption spectrum, extinction coefficient, and PL can be tuned by changing sizes of the QDs (along with chemical composition), they are thought beneficial for light harvesting and solar cell applications.

In the breaking of bulk semiconductors into QDs, the intra-atomic bonds are broken and result in the formation of more edge atoms and unsaturated bonds on the surface. These properties thus offer a higher surface activity which increases the catalytic activity. Because of the improved catalytic activity, QDs are therefore beneficial for use in HERs, bioimaging, and electrochemical and colorimetric sensing. So far QDs of MoS_2_ and graphene have successfully been used for the electrochemical and colorimetric detection of different biomolecules like proteins, DNA, RNA, and living cells as well as for the detection of different gases (NH_3_, H_2_S, etc.) and metal ions (Ag, Hg, etc.). To date, several reports have been published on the colorimetric detection of H_2_O_2_ by MoS_2_/graphene QDs-based sensors [67,68].

### 3.4. Fabrication of QDs

**Figure 6:**
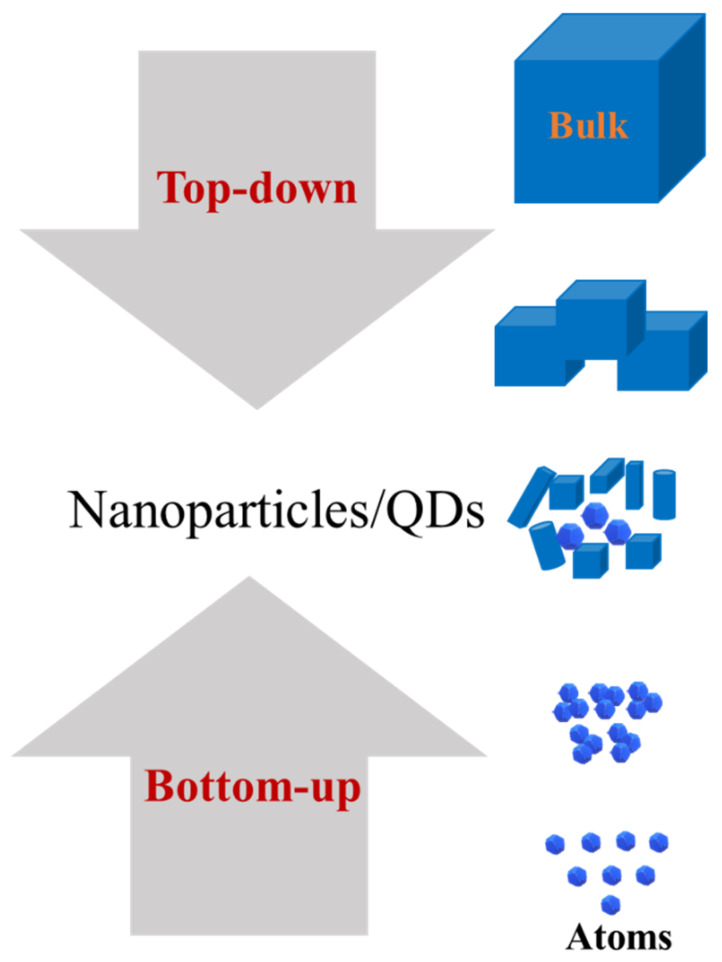
Schematic representation of the ‘top-down’ and ‘bottom-up’ strategies for fabrication of QDs.

Synthesis strategies followed for the MoS_2_ QDs fabrication are generally categorized into two main groups, namely top-down and bottom-up fabrication techniques. In ‘top-down’ techniques bulk material is broken into pieces of the ‘desired sizes’ while in ‘bottom-up’ techniques atoms and molecules are linked together to make the nanocrystals of ‘desired size’. Figure 6 shows the schematic illustration for both approaches called top-down and bottom-up.

#### 3.4.1. Bottom-Up

The bottom-up synthesis of MoS_2_ QDs involves atom-by-atom assembling of respective anions to produce nanostructured particles of MoS_2_. The respective anions are provided by the molybdenum and sulfur precursors. The prominent techniques among the bottom-up exfoliation methods include sol-gel, atomic layer deposition (ALD), hydrothermal synthesis, and chemical vapor deposition (CVD). For a detailed understanding of the bottom-up approaches, the reader is encouraged to consult several reviews [53,69,70,71] found in the literature. In the current review, we will only focus on the top-down approaches used for the exfoliation of MoS_2_ QDs.

#### 3.4.2. Top-Down

The ‘top-down’ fabrication is used for thinning the bulk MoS_2_ into nanocrystals or quantum dots by chemical or physical methods. These exfoliation methods are mechanical, sonication-assisted, ion-intercalation, electrochemical and electro-Fenton reaction, microwave-assisted, laser-assisted, and cryo-mediated exfoliation processes. A detailed description of these exfoliation methods is given below.

##### Mechanical Exfoliation

Micromechanical cleaving or Scotch Tape [72] method has effectively been used for the exfoliation of graphene and monolayers of 2D LTMDs.

Grinding-assisted exfoliation of MoS_2_ QDs is another example of a mechanical exfoliation technique. In grinding exfoliation, shear forces peel single and few layers from bulk MoS_2,_ and the detached layers are then broken into small pieces by sonication [73,74]. For example, Yao et al. [73] prepared monolayer and few-layer nanosheets by a less-energetic shear exfoliation process from bulk MoS_2_ crystal. The exfoliation process includes wet grinding of MoS_2_ crystal in N-methyl-2-pyrrolidone (NMP) followed by sonication in a 45-vol% ethanol/water solution. NMP was used as an effective surfactant to avoid the retaking of the exfoliated nanosheets. Organic solvents, such as dimethylformamide (DMF) and dimethyl-sulphoxide (DMSO) resulted in a lower concentration of the exfoliated nanosheets in the respective aqueous solutions. The developed method successfully produced nanosheets of lateral sizes 20–60 nm and thickness of 1.2–8.5 nm.

**Figure 7:**
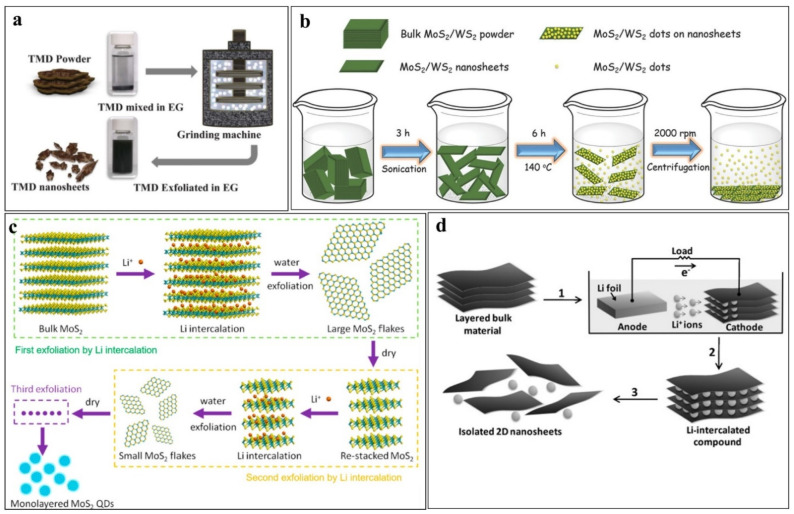
Exfoliation of MoS_2_ QDs by: (**a**) wet-grinding (Reproduced with permissions from [74]; (**b**) ultrasonication-assisted (Reproduced with permissions from [18]. Copyright WILEY-VCH Verlag GmbH & Co. KGaA, Weinheim, 2015); (**c**) ion-intercalation (Reproduced with permissions from [75]. Copyright Elsevier B.V., 2015); and (**d**) electrochemical exfoliation (Reproduced with permissions from [76] under a Creative Commons license).

Ibrahem et al. [74] exfoliated MoS_2_ QDs by a ball-milling assisted wet grinding process, schematically shown in Figure 7a. In the grinding process, bulk MoS_2_ powder was mixed with pure ethylene glycol (EG, wt%: 1) and zirconia beads (100 µm, density >5.95 g cm^−3^) in a ball mill at a constant rotating speed for 8 h. In the grinding process, the impact and friction forces peel smaller pieces whereas the shear forces exfoliated single or few-layer sheets. The microscopic observations revealed that extending the milling time beyond 8 h, has a negligible effect on the thickness of the exfoliated sheets. Few-layer MoS_2_ nanosheets with lateral sizes of several hundred nm and good dispersibility were exfoliated.

##### Ultrasonic Exfoliation

In ultrasonic exfoliation processes, sonication of bulk MoS_2_ powders (dispersed in different solvents) is carried out. The energetic ultrasonic waves initiate micromechanical acoustic cavitation processes, that can break the inter/intra-layer bonds of MoS_2_ sheets. The ultrasonic cavitation produces a series of micro-mechano-chemical effects such as microjets, shear forces, shock waves, and pressure cavities due to pressure and temperature variations in the colloidal solution. These effects carry enough energy to break the inter/intra-layer bonds and isolate monolayers and QDs. In a typical ultrasonication-assisted exfoliation process, Xu et al. [18] continuously sonicated bulk MoS_2_ powder dispersed in DMF for 3 h. The mixture was refluxed at 140 °C for 6 h and centrifuged at 2000 rpm to separate the QDs as supernatant. An illustration of the following exfoliation procedure is shown in Figure 7b. The exfoliation process could not produce QDs when DI water and acetone were used as solvents to disperse the MoS_2_ powders. Many other solvents, including H_2_SO_4_ [77], DMF [18,78,79], NMP [80], DMUE [18], isopropyl alcohol (IPA) [81], and ethylene glycol [82,83] have been successfully used for the dispersion of bulk powders in the sonication-assisted exfoliation of MoS_2_ and other 2D materials.

##### Ion-Intercalation Exfoliation

In ion-intercalation exfoliation, ions such as Li^+^, K^+^, and Na^+^ are employed to isolate monolayers of 2D materials. These ions can easily intercalate between the layers of 2D materials due to large inter-layer spacing (e.g., MoS_2_: ~0.65 nm [84]). Intercalation of the ions weakens the layer-layer interactions and makes the isolation of the monolayers easy. Ion-intercalation process usually starts by soaking bulk MoS_2_ powders with source compounds and salts of the Li^+^, K^+^, or Na^+^ ions. The soaking normally lasts for several hours, so that the ions can insert well between the layers and produce the ion-intercalated precursor material, i.e., lithiated MoS_2_ or Li_x_MoS_2_ in a typical Li^+^-intercalation exfoliation process of MoS_2_. These ion-intercalated samples are then sonicated to exfoliate and centrifuged to separate the exfoliated material. The excessive ions and reagents are usually washed out of the exfoliated material by dialysis.

To produce the Li_x_MoS_2_, Qiao, et al. [75] reported that MoS_2_ soaked powder in an argon-filled bottle stored for two days which is further filled with *n-butyl lithium* (*n*-BuLi) and hexanes solution. A schematic representation of the multi-step Li^+^-intercalation process is shown in Figure 7c. The Li_x_MoS_2_ retrieved by filtration and repeatedly washed with hexanes was dispersed in water and sonicated. Later on, to flocculate and neutralize the pH, HCl was poured into the sonicated sample. The flocculate was then centrifuged several times and vacuum-dried repeatedly. Excessive lithium hydroxide was removed by dialysis against DI water and finally, it was centrifuged and annealed at 90 °C in a bath sonication. The obtained QDs were of ~3.0 nm and ~1.0 nm in lateral sizes and thickness, respectively.

Similarly, Zhou, et al. utilized Na^+^ ions intercalation to exfoliate MoS_2_ [85]. In the following exfoliation process, MoS_2_ mixed with Na sand was kept in a vacuum at 160 °C for 12 h in a sealed Pyrex tube. The intercalated sample was then exposed to ambient air for an hour and sonicated for 30 min in 100 mL water. To separate the exfoliated QDs and large-sized nanosheets from the un-exfoliated MoS_2_ powder, the colloidal solution was filtered followed by centrifugation of the filtrate at 7000 rpm for 15 min. Excessive Na ions were removed by dialysis of the supernatant for 3 days against DI water. The yield of QDs was about 11% for the reported method.

In another experiment, Ali et al. [25] dispersed MoS_2_ powder and NaOH in ethanol by energetic stirring for 30 min. Later on, The dispersion was heated at 180 ℃ for 12 h in a 50 mL Teflon-lined autoclave reactor. The intercalated mixture was cooled to room temperature and DMF was added to it. The resultant solution was continuously sonicated for 3 h in an ultrasonic bath at room temperature and centrifuged to separate the exfoliated QDs as supernatant. The obtained supernatants were dialyzed in a 1000 Da dialysis tubing for 48 h against DI water to remove excessive NaOH and solvents. MoS_2_ QDs with 3.7 nm and 1.2 nm of average lateral sizes and thickness were produced by this reported method. The final production yield for QDs was about 20% with a significant improvement in the production yield, compared to some previous methods [18,85].

##### Electrochemical Exfoliation

Gopalakrishnan et al. [16] proposed a new prominent mechanism for electrochemical synthesis of MoS_2_ QDs analogous to posed mechanism used for electrochemical exfoliation of graphene nanoribbons and QDs. In this technique, the initial cleavage was done by the radicals triggers which result in the exfoliation of the material. By applying the DC voltage to the highly diluted electrolytes, two radicals called hydroxyl and oxygen are formed. Similarly, the MoS_2_ anodes swell because of the incorporation of anions called TFSI –*(bis(trifluoromethane)sulfonimide)* and Cl^−3^, while the electrolyte dissolves MoS_2_. hydroxyl

The electrochemical etching, proposed by Gopalakrishnan et al. [16] can be used for a desirable size of MoS_2_ QDs, where the commercially existing powder of MoS_2_ in disc form with a diameter of 1 cm was used. Further, across these MoS_2_ discs, a constant DC voltage in the presence of *Lithium bis(trifluoromethanesulfonyl)imide* (LiTFSI) or *1-Butyl-3-methylimidazolium chloride* ([BMIm]Cl) was applied and collected after three hours for the centrifuge. Interestingly, the particles obtained by this technique is depending upon the concentration. For example, LiTFSI solution, at concentrations of 0.1 and 1 wt%, provided particles with sizes of 2.5 and 4.6 nm, respectively while [BMIm]Cl yielded larger particles with sizes of ~2.8 and ~5.8 nm. In the two exfoliation processes, the same concentrations of LiTFSI and [BMIm]Cl were used.

Zeng et al. [76] exfoliated MoS_2_ by a lithiation-assisted electrochemical process. In the reported process, shown in Figure 7d MoS_2_ was incorporated as the cathode in an electrochemical setup and Lithium foil was used as the anode to supply lithium ions. After lithium intercalation, the lithiated MoS_2_ was sonicated to exfoliate and detach a single or few layers. In the Li-intercalation-assisted electrochemical process, lithium plays dual roles. First, the Li^+^ions intercalate between the layers, expanding the interlayer distance and weakening van der Waal’s interaction between the layers. Second, metallic Li, produced due to the reduction of the intercalated Li^+^ ions, reacts with water to form LiOH and H_2_ gas, which pushes the layers further apart. Sonication of the lithiated material detaches the thinner nanosheets from the bulk MoS_2_. The Li^+^ intercalation-assisted electrochemical exfoliation produced monolayer nanosheets and this method can be scaled-up by increasing the size of the electrodes.

##### Electro-Fenton Assisted Electrochemical Exfoliation

Fenton reagents (Fe^2+^ and H_2_O_2_) are powerful oxidation etchants, primarily used for the degradation of environmental pollutants by the generation of highly reactive hydroxyl radicals [86]. Based on the generation of the Fenton reagents, the Fenton reaction can be conventional, photo-induced, or electrochemically induced Fenton reactions. Electrochemically induced Fenton (electro-Fenton) reaction, is an advanced oxidation reaction, where H_2_O_2_ is produced in-situ at the cathode, by reduction of oxygen as follows:

O_2_ + 2e^−^ + 2H^+^ → H_2_O_2_ (1)

where, the addition of ferrous ions (Fe^2+^), enhances the oxidation activity by the generation of hydroxyl radicals (^−^OH), as shown by the following chemical reaction:

Fe^2+^ + H_2_O_2_ + H^+^ → Fe^3+^ + OH + H_2_O (2)

and  Fe^3+^ + e^−^ → Fe^2+^ (3)

The Fe^2+^ ions are re-generated at the cathode, resulting in the production of OH radicals in a catalytic and controlled manner, making the electro-Fenton reaction tunable and controllable.

**Figure 8:**
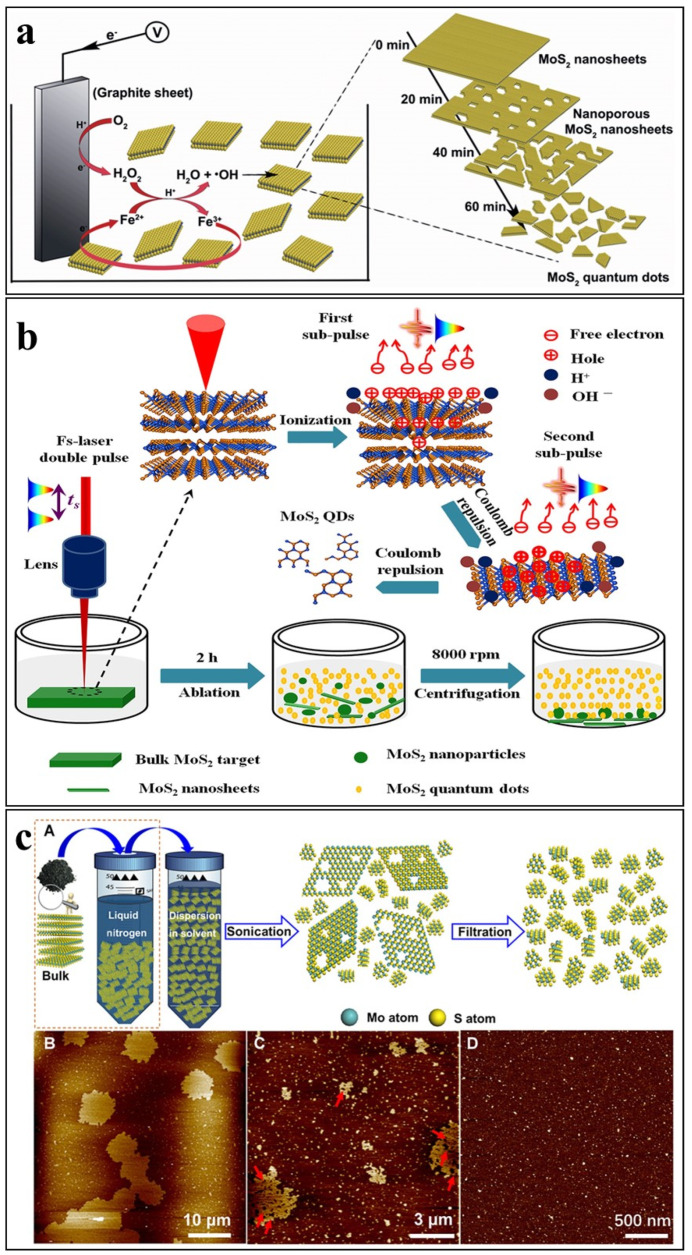
Exfoliation of MoS_2_ QDs by: (**a**) electro-Fenton (Reproduced from [17], with permissions from Royal Society of Chemistry); (**b**) Laser ablation (Reproduced with permissions from [23] under a Creative Commons license); and (**c**) Cryo-mediated exfoliation (From [22] with permissions from AAAS).

To synthesize the graphene-oxide and MoS_2_ QDs, Li et al. [17] developed a technique (shown in Figure 8a) where they used the electro-Fenton reaction. In this technique, besides the addition of MoS_2_ and FeSO_4_ to the electrolytes, a solution is also added. Then slowly and gradually sulfuric acid was added to control the pH. Later on, under a potential of −0.5 V, the solution was saturated by using compressed air and O_2_. A magnetic bar stirrer was also used not only for continuous stirring but for mass transfer. Then, after one hour, to collect the products and remove the impurities, the dialyzing of sulfuric acid has done. To ensure the removal of impurities, an additional day and use of ultrapure water will confirm the hydrolysis of water technique. Interestingly, the lateral size of 3–8 nm and thickness ranging up to 2 nm further confirms the bi- and mono-layer feature of the QDs.

##### Microwave-Assisted Exfoliation

Among different energy resources, microwaves work differently, because it induces ultrasonic waves under pressure using mechanical and plasma techniques. It enhances the efficiency of the chemical reaction by producing the hotspots which result in fast heating with spin and charge polarization. Microwaves homogenously heat the entire reaction solution and provide the necessary reactions evenly for the nucleation center [87]. In microwave-assisted exfoliation, the reaction efficacy is mainly based on the absorption capacity of the precursors and the solvents involved in the reaction [69].

In their pioneering work, Lu et al. prepared pristine and histidine-doped MoS_2_ QDs from commercial MoS_2_ nanoflakes [62] by microwave-assisted exfoliation. In their experiment, MoS_2_ nanoflakes in H_2_O_2_/ethanol solution were stirred and heated in a microwave tube (2445 MHz) at 200 °C for a given period. After heating, the solution was allowed to cool and settle over a day and then filtered through a syringe filter of pore size 0.22 µm. The filtered solution was further purified by dialysis for 2 days. Histidine-doped QDs were fabricated by following the same procedure except adding L-histidine powder to the MoS_2_ nanoflakes and H_2_O_2_/ethanol solution before heating with the microwaves. The exfoliated MoS_2_ QDs were of ~4.2 nm in lateral dimensions.

##### Laser Ablation

In laser ablation of MoS_2_ and other 2D layered materials, the irradiated femtosecond laser pulses cause photo-induced ionization at the surface of the target materials. The electron ejection produces Coulumbic repulsive forces between the layers of the target material. As a result, the inter-layer interactions are weakened, which in turn detach the single and few layers from the bulk materials. In laser ablation processes, the target MoS_2_ is immersed in a solvent and continuously rotated while irradiating with the laser pulses.

Li et al. [23] reported the exfoliation of MoS_2_ QDs by a temporally shaped femtosecond laser. In the reported laser ablation process, the conventional femtosecond laser beam was split into two subpulses with energy ratios of 1:1 and total laser fluence of 0.77 Jcm^−1^. The time delay between the two sub-beams was ~0–10 ps. The two subpulses have a large total fluence as compared to the ablation threshold of MoS_2_ (0.24 J cm^−1^). The proposed laser ablation process is a multi-level photoexfoliation of MoS_2_ and water photoionization-enhanced light absorption. The reported laser ablation process is shown in Figure 8b, where the first subpulse ejects thermal electrons from the MoS_2_ surface and creates coulomb repulsion between the layers. This electrostatic repulsion consequently reduces the inter-layer interactions and triggers the first-level photoexfoliation. The second sub-pulse irradiated with 10 ps delay causes enhance coulomb repulsion due to enhanced ionization-induced charge accumulation on the ablated or bulk MoS_2_ surface, causing the second level of photoexfoliation. Meanwhile, the first subpulse ionizes water molecules and increases the local charge density. The enhanced local charge density consequently enhances the light absorption for the second pulse, which improves the production yield for exfoliation of the MoS_2_ QDs. In the case of a single pulse femtosecond laser, the total fluence (0.77 J cm^−1^) of the single pulse is much higher as compared to the MoS_2_ ablation threshold (0.24 J cm^−1^) and thus causes thermal phase-change mechanisms including melting and evaporation, therefore causing the detaching of larger sized nanosheets and nanoparticles. The temporally shaped laser beam was normally focused on a rotating MoS_2_ target immersed in DI water. After ablation of 2 h in ambient air temperature and pressure, the aqueous solution was allowed to settle for a maximum of 4 h and centrifuged to separate the exfoliated QDs as supernatant. The supernatant was further sonicated to avoid any agglomeration of the QDs. The laser ablation successfully exfoliated ultra-small single-layered MoS_2_ QDs with average sizes of about 2.6 nm. The developed procedure can also be carried out in other aqueous media such as NMP.

Yanmin et al. [26] exfoliated MoS_2_ QDs by a sonication-assisted femtosecond laser ablation process. In the reported strategy, precursor MoS_2_ powder was dispersed in NMP and irradiated by a femtosecond laser pulse with laser power of 400 mW and pulse duration of 80 fs, for 30 min. During laser injection, the powder was stirred by a magnetic stirrer bar to avoid gravitational settling of the bulk powder. The laser ablation exfoliates few-layered sheets from the bulk particles, which are separated by centrifugation. After laser irradiation, the separated supernatant was sonicated for a given period to break into smaller QDs. The developed method was also used to fabricate QDs of WS_2_ and hBN.

##### Cryo-Mediated Exfoliation

Cryo-mediated exfoliation of MoS_2_ and other 2D layered materials usually comprises treating the initial bulk material at cryogenic temperatures by soaking it in liquid nitrogen. This treatment results in a significant temperature change in the material and the fast-thermal quenching form small cracks in the material. These cracks act as capillaries for solvent permeation [88] that facilitate the exfoliation of the material by bath sonication.

Wang et al. introduced cryo-mediated exfoliation for MoS_2_ and other LTMDs [22]. The developed exfoliation procedure is a two steps process. The first step includes the pre cryo- treatment of the bulk MoS_2_ powder by soaking it in liquid nitrogen for some time (10 min to several hours). Similarly, in the next stage, the cryo-treated powder is immediately dispersed in a solvent (IPA/H_2_O, 1:1) and exfoliated by bath sonication. In the case of all 2D materials, an initial concentration of ~3 mg ml^−1^ of the dispersions was used. To separate the exfoliated nanostructures from the unexfoliated bulk material, the colloidal dispersion was centrifuged at 6000 rpm for 30 min and the supernatant was collected by pipette, following the cryo-mediated exfoliation and fracturing treatment. The collected supernatant was vacuum filtered through ultrafine membranes (pore size: ~25 nm, VSWP02500, Millipore, Houston, TX, USA) to separate the QDs from the mixed dispersions containing QDs and the exfoliated nanosheets. A schematic illustration of the cryo-mediated exfoliation is shown in Figure 8c (A), whereas B, C, D show AFM images of the exfoliated nanosheets and QDs obtained by the exfoliation process.

### 3.5. Advantages and Limitations

The most commonly used techniques for the fabrication of MoS_2_ QDs include mechanical and grinding exfoliation, electrochemical etching, liquid exfoliation, electro-Fenton treatment, ion-intercalation exfoliation, laser ablation, and cryo-mediated exfoliation. All of these synthesis strategies, i.e., mechanical, grinding, ultrasonic-assisted, electrochemical, electro-Fenton, hydrothermal, laser ablation, and cryo-mediated exfoliation [16,17,18,19,20,21,22,23,24], have their advantages and limitations, summarised in Table 2.

For example, mechanical exfoliation is a simple, scalable, green, and inexpensive, method for exfoliation of MoS_2_ nanosheets and QDs that can be used for fabrication as film or composites [89]. However, it has limitations of (1) producing smaller structures with the characteristic of QDs, (2) needing large-sized TMD crystals to prepare nanosheets of suitable size for characterization and device fabrication, and (3) usually producing flakes of nonuniform thickness [90]. Sonication-assisted exfoliation is the only reliable method for mechanical exfoliation of MoS_2_ QDs, but it is a very tedious and laborious process. Similarly, grinding exfoliation may produce large, polydisperse, and multi-layered particles.

Good productivity, ease of operation, economic and environmental feasibility, and mild synthetic conditions are listed as some of the advantages of liquid exfoliation and hydrothermal synthesis techniques [91]. The ion-intercalation exfoliation can yield MoS_2_ QDs with excellent hydrogen evolution reactions (HER) catalytic activity [75]. However, they also have some limitations such as the ion intercalation-assisted liquid exfoliation strategies may be tedious and time-consuming because of the lengthy procedures, and hazardous as they require toxic organic solvents.

Electrochemical exfoliation demonstrated excellent results in terms of the quality of the exfoliated flakes, high production yields, and lower environmental impact. Electrochemical etching is a valid alternative to ion-intercalation-assisted exfoliation in organic solvents for catalytic applications as well as other applications where the 2H semiconducting characteristics are required [92]. The main limitations in electrochemical etching are the harsh conditions [93], complicated chemical processes [94], sophisticated electrochemical equipment as well as multiple steps with post-process cleaning to remove by-products.

The pulsed laser ablation route for exfoliation of MoS_2_ is a versatile, simple, economical, and promising method to obtain highly pure colloidal suspensions of QDs and nanosheets. Laser properties such as wavelength, intensity, pulse width, and spot size, as well as the thermal and physical properties of the target and liquid, are taken into account as effective parameters for controlling the characteristics of the exfoliated material [95]. Despite the successful exfoliation of the MoS_2_ nanosheets, the synthesis of large and uniform single crystal MoS_2_ nanosheets is still a challenging issue, limiting further applications of the pulsed laser ablation method.

The cryo-mediated exfoliation of MoS_2_ QDs simply relies on the temperature-induced expansion and contraction, nitrogen intercalation, and gasification of the liquid nitrogen. Cryo-mediated exfoliation has the advantages of high efficiency (short sonication time and ultrathin nanosheets), environmental friendliness (non-hazardous medium, and solvents), and no introduction of additives. Some common crucial issues regarding these strategies are (1) their effective and efficient separation into mono/few layers so that no damages are breaking happen to the sheets, (2) prevention of the separated layers from re-stacking and re-aggregation, and (3) simple and efficient separation of the exfoliated nanostructures from the unexfoliated material. The scientific community requires more creative efforts and expansive resources, to overcome these constrictions and establish simple, scalable, and inexpensive fabrication methods for the exfoliation of MoS_2_ QDs.

## 4. Applications

Bulk MoS_2_ has traditionally been employed for solid lubrication. Although MoS_2_ has a relatively lower thermal stability and hence maximum operating temperatures, in high-vacuum applications it can be considered a replacement, for graphite. Being expansively studied, for more than a decade, a few interesting implementations in various technological fields have been found, a few of which are briefly discussed here.

### 4.1. Field Effect Transistors (FETs)

MoS_2_ has a significant bandgap with suitably large carriers mobilities, due to which it can be potentially employed in FET-related applications. For example, some studies related to the monolayer MoS_2_ QDs FETs, have shown its promised performance and reported carrier’s mobility values of about 0.21 cm^2^V^−1^s^−1^ with an on/off ratio of ~10^5^ [37] comparable to the monolayer MoS_2_ devices [4] Although MoS_2_-FETs mostly expresses n-type features, scientists are hopeful that they will show better performance than the commonly used Si-FETs in specific conditions, including on/off ratios and power efficiencies [96]. Some efforts have already been made for improving the MoS_2_-FETs using strategies such as understanding the ambipolar transports, enhancing electrical injection, and reducing substrate interactions [7,97,98].

### 4.2. Photodetection

Because of the band-gap, MoS_2_ has offered itself to optoelectronics and a recent study related to the fabrication of a photodetector based on monolayer MoS_2_ has shown reasonable photo-sensitivity of about 880 AW^−1^ with a broadband photo-response, in the range 400–680 nm [99] while the sensitivity rises by 104 times on employing the monolayer MoS_2_ in a hetero-structure with graphene [93]. Recent studies have shown that phototransistors based on the hybrids of 2D materials have great detection sensitivity and wide wavelength response, offering possibilities for cutting-edge photodetector technology. For example, Ulaganathan et al. [100] have reported a high-performance broadband photodetection device using the MoS_2_ QDs/InSe hybrid structure as the conducting channel.

### 4.3. Photovoltaics

Having optical absorbance in the visible-light range and higher as compared to Si, single-layer MoS_2_ lends itself as a potential employer in photovoltaics. Studies related to its hetero-structures with graphene and single layer WS_2_ have already resulted in photo-conversion-efficiencies (PCE) ~1% [101]. Although the reported PCEs are low, the resulting power densities are ~104 times better than Si devices as the active-surface area in these devices (~1 nm) is extremely small compared to those for Si-based devices (~100 micrometers). More than 5% PCE has already been achieved for a type-II heterojunction solar-cell fabricated of p-doped Si with single-layer MoS_2_ grown by the CVD [102].

Although the semiconducting nature of the abundant 2H-MoS_2_, confines its capability of use as an electrode, it can be converted into the 1T-phase with 107 times higher conductivity than the 2H-phase [103]. MoS_2_ has been shown better performance as compared to graphene in terms of energy and power densities. The MoS_2_ QDs fabricated by laser ablation and cryo-mediated exfoliation have a highly crystalline 2H phase. These QDs can hinder the performance of the photovoltaic devices and hence should be replaced with MoS_2_ QDs prepared by other suitable techniques.

### 4.4. Sensing

In sensing applications, it is noteworthy, that single-layer MoS_2′_s PL greatly changes by adsorbing oxygen and water where an electron transferred from the MoS_2_ surface to the H_2_O/O_2_, stabilizes the exciton and causes a 100x increase in the PL intensity [104]. Experiments related to the detection of NO, NO_2_, NH_3,_ and humidity by monolayer MoS_2_ FET-sensors, revealed their unstable electrical nature but it can be overcome with the use of few-layer MoS_2_ [16,105]. it has already recorded a sensitivity of <1 ppm for the case of NO detection. MoS_2_ shows brilliant catalytic activities along with long-term stabilities, making it highly favorable for applications as a catalyst. Moreover, the reduced sizes of MoS_2_ QDs (almost about <10 nm) attributed to extremely large specific surface areas, active edge effects, and intensive quantum confinements greatly induce its potential as a catalyst. Therefore, it has shown good enzyme-memetic applications for different nano-structures such as nano-flowers, nano-flakes, and nano-particles [106]. Ion-intercalation exfoliation is useful in enhancing the PL properties of the exfoliated MoS_2_ [18], hence MoS_2_ QDs fabricated by ion-intercalation can be useful in fluorescence-based sensing and bioimaging. MoS_2_ QDs fabricated by electrochemical exfoliation have excellent catalytic properties and abundant active edges that can be actively used in sensing applications.

### 4.5. Composites and Sieving Membranes

Monolayer nanosheets and QDs of MoS_2_ have excellent mechanical strength, high electrical and thermal conductivities, enhanced catalytic activity, better biocompatibility, and non-toxicity. Recent studies showed that monolayer MoS_2_ has the potential for fabrication of a highly selective, and permeable separation membrane with better performance for water treatment and purification compared to inorganic filtration or a polymer-based membrane. Single layer MoS_2_ QDs with tunable functionalities and enhanced surface properties facilitate the membrane modification as a trade-off between the permeability and selectivity properties [107,108].

Although significant progress has been made on the separation mechanisms of graphene and graphene-based materials, their great potential for liquid and gas separation applications has been demonstrated. However, graphene membranes, have some inherent limitations such as poor dispersion, high cost, and large-scale production. Further, long-term stability, resilience to backwashing and chemical cleaning, and effective layer separation of graphene membranes are major concerns in their commercialization. The incorporation of graphene layers between MoS_2_ monolayers increases the layer spacing from 0.62 nm to 1.16 nm [109], as GO-based separation membranes with pore sizes of 1.3 nm [110] had successfully been used in the separation of ionic and molecular solutions. MoS_2_ has a relatively high elastic modulus (200–300 GPa) compared with GO (207.6 ± 23.4 GPa) which further enhances by ~3% for MoS_2_ monolayer sandwiched between graphene layers [111]. MoS_2_ has shown better performance in the separation of ionic species as compared to graphene, phosphorene, BN, and MoSe_2_ [108]. LTMDs-based multifunctional polymer composite gels with tunable optical and mechanical properties can be useful in various applications such as biosensors, cartilage, tissue repairing, drug delivery, and wound dressing. The properties of these gels are easily tuned by the amount of the MoS_2_ and other LTMDs [112].

In water purification and treatment applications, biocompatibility and nontoxicity are the most important features, therefore the appropriate choice could be the MoS_2_ QDs and nanosheets exfoliated by the cryo-mediated exfoliation process.

## 5. Conclusions

MoS_2_ QDs have already shown gigantic potential in numerous applications, ranging from electronic to photovoltaic, catalysis to photodetection, sensing to biosensing, and polymer composites to water treatment and purification. The application range of the MoS_2_ QDs is still expanding, and new potential fields of applications are explored with creativity in their functionalization and usability. The photovoltaic and water purification applications are still in their infancy, and it can be expected that the use of MoS_2_ QDs heterostructures and hybrids would benefit these areas. Similarly, for advances in stretchable wearables and wound dressings, the ideas related to enhancing the dispersibility of MoS_2_ in different materials can be beneficial. All these applications and employability essentially require a scalable, green, and inexpensive method for the exfoliation of bulk MoS_2_ into monolayer nanosheets and QDs.

Among the current existing top-down strategies for fabrication of MoS_2_ QDs, laser ablation and cryo-mediated exfoliation techniques look great in terms of non-hazardous environmentally friendly nature. For good production yield, ion-intercalation exfoliation looks more convincing procedure, whereas ultrasonic and mechanical exfoliation are used as simple techniques. However, as discussed, all these existing exfoliation processes still require a lot of attention and hard work for the commercial and scalable production of MoS_2_ QDs with specific size control. Ideas and advances in 2D materials exfoliation research will not only advance science, engineering, and technology but can be considered as a field of prospective research in the coming days.

## Figures and Tables

**Table 1:** 2D Family.

Graphene Family	Graphene		hBN	BCN	Fluorographene	Graphene Oxide
2D Chalcogenides	MoS_2_, WS_2_, MoSe_2_, WSe_2_		Semiconducting dichalcogenides: MoTe_2_, WTe_2_, ZrSe_2_/S_2_, etc.	Metallic dichalcogenides; NbSe_2_/S_2_, TaS_2_, TiS_2_, NiSe_2_ etc.		
				Layered Semiconductors; GaTe/Se, Bi2Se3, InSe, etc.		
2D Oxides	Micas BSCCO	MoO_3_, WO_3_	Perovskites: LaNb_2_O_7_, (Ca/Sr)_2_Nb_3_O_10_, Bi_4_Ti_3_O_12_, Ca_2_Ta_2_TiO_10_ etc.	Hydroxides: Eu(OH)_2_, Ni(OH)_2_, etc.		
	Layered Cu oxides	TiO_2_, V_2_O_5_, TaO_3_, RuO_2_, etc.		others		
Key						
	Stable materials in ambient conditions			Potentially stable materials in ambient conditions		
	Stable materials in inert conditions only			3D compounds- have been exfoliated into monolayers		
Others: includes borides, nitrides, carbides, etc., have been or can be isolated, such as BCN, boron carbon nitride						

**Table 2:** A comparison of different exfoliation strategies.

Precursors	Method	Size (nm)	Yield (%)	Notes	Ref.
MoS_2_ powder Organic solvents	Mechanical exfoliation	~10–60	-	Simple, green, and cost-effective Low production yield Multi-layered QDs	[65,66,67]
MoS_2_ powder Solvents	Ultrasonic Exfoliation	~2.5	~10	Facile, inexpensive Commonly used exfoliation process for LTMDs Can be carried out in several solvents	[18,68,69,70,71]
MoS_2_ Li, K, Na salts (LiOH, etc.) Organic solvents (DMF, etc.)	Ion-intercalation Exfoliation	~4	10–20	Small-sized QDs, good yield Laborious washing processes High catalytic activity	[25,76]
MoS_2_ Electrolyte solvents (LiTFSI, [BMIm]Cl, etc.)	Electrochemical Exfoliation	~3–6	-	Small, monolayered QDs can be obtained Tedious post-process washing Harsh reaction conditions	[16,77]
MoS_2_ Fenton reagents	electro-Fenton Reaction	~5	-	A good alternative to intercalation and electrochemical exfoliation processes	[17]
MoS_2_ DMF, ethanol	Microwave-assisted Exfoliation	~4.5	-	Low production yield Depends on the microwave absorption ability of the solvent	-
MoS_2_ DI water, NMP	Laser Ablation	~3.0		Green and facile, produce pure QDs and nanosheets MoS_2_ powder and pellets can be used Produce poly-crystalline exfoliated nanostructures	[23,81]
MoS_2_ Liquid Nitrogen	Cryo-mediated Exfoliation	~2.5	1	Simple, environmentally friendly No by-products Low production yield	[22]

## Data Availability

Not applicable.
